# Assessing the impact of removal scenarios on population viability of a threatened, long-lived avian scavenger

**DOI:** 10.1038/srep16962

**Published:** 2015-11-23

**Authors:** Antoni Margalida, Mª Àngels Colomer, Daniel Oro, Raphaël Arlettaz, José A. Donázar

**Affiliations:** 1 Department of Animal Production (Division of Wildlife), Faculty of Life Sciences and Engineering, University of Lleida, 25198 Lleida, Spain; 2 Division of Conservation Biology, Institute of Ecology and Evolution, University of Bern, Baltzerstrasse, 6 3012 Bern, Switzerland; 3 Department of Mathematics, Faculty of Life Sciences and Engineering, University of Lleida, 25198 Lleida, Spain; 4 Population Ecology Group, Institut Mediterrani d’Estudis Avançats IMEDEA (CSIC-UIB), Miquel Marques 21, E-07190 Esporles, Spain; 5 Swiss Ornithological Institute, Valais Field Station, Rue du Rhône 11, 1950 Sion, Switzerland; 6 Grupo de Investigaciones de la Biodiversidad, IADIZA, CONICET–CCT, Av. Ruiz Leal, Parque General San Martín, Mendoza, Argentina; 7 Department of Conservation Biology, Estación Biológica de Doñana, CSIC. Avda de Américo Vespucio s/n, Isla de la Cartuja, E-41092, Sevilla, Spain

## Abstract

The removal of eggs or chicks from wild populations to create captive populations, reinforce free-ranging populations or reintroduce species into the wild is a restoration tool that requires an assessment of potential detrimental effects upon the donor population. This is an absolute prerequisite when wild donor populations are scarce and small. Here, we forecast the population trend of the largest European population of the bearded vulture (*Gypaetus barbatus*) over the next 30 years under different demographic and management scenarios (removal of eggs, chicks or fledglings). Projections derived from the combination of a PDP model (Population Dynamic P-system) and a Box-Behnken design would lead to a decline in 77% of all 57 scenarios analysed. Among the 13 scenarios predicting a population increase, only 4 seem realistic in terms of growth rate (0.04%–1.01%), at least if current age at first breeding and productivity would remain constant over time. Our simulations thus suggest that most extraction scenarios would have detrimental effects on the demography of the donor population. Release of captive-born young or removal of only the second hatched chick for subsequent captive rearing and translocation into the wild appear to represent much better supplementation and reintroduction options in this threatened species.

The conservation of threatened species requires reliable and robust assessments of their long-term demography so as to envision appropriate management. Today, the factors regulating demographic processes in wildlife are largely influenced by anthropogenic activities^1^,^2^,^3^, which calls for strategies to mitigate these impacts. Identifying these factors and the vital parameters most affected by them is essential to predict population trajectories and develop targeted and efficient management^4^,^5^. Population viability analysis (PVA) is a useful modelling tool for managers and conservationists to assess population demography, guide conservation action and prioritize resource investment^6^,^7^,^8^. Nevertheless, this tool has obvious limitations due to the inherent shortcomings of usually limited demographic information^9^ and the difficulty in identifying causes of population declines^6^,^10^. Despite these drawbacks, PVA models are a widespread tool among managers and policy makers who often need to make quick decisions based on sensible demographic predictions^1^,^4^,^6^,^11^,^12^,^13^,^14^,^15^,^16^.

New computational models (Population Dynamic P-system, PDP models) inspired by cell properties have been used to assess the population dynamics of threatened populations in relation to food availability and climate change^17^,^18^,^19^, providing reliable alternatives to traditional PVA models. PDP models may account for environmental stochasticity and have the advantage that modelling can be flexibly adapted to the various biological, technical and methodological specificities of a given demographic study, and, typically, to the scope and wishes of the modeller^20^. This flexibility allows, for instance, to incorporate into the modelling process correlations between demographic parameters (which occur in most populations) more easily than with conventional PVA models which have largely ignored this aspect^21^.

The largest European population of bearded vultures (*Gypaetus barbatus*), with 152 breeding territories (83% of the EU population), lives in the Pyrenees (Spain and France). Due to its small size and peripheral situation with respect to species’ main range^22^,^23^, the Pyrenean population may be particularly prone to demographic and environmental stochasticity, i.e. to extinction^24^,^25^,^26^. The Pyrenean bearded vulture population is characterized by both a long-term reduction in breeding output partially due to density-dependent regulation^27^, and a relatively high adult mortality rate, probably as a consequence of ingestion of poison baits and lead from ammunition fragments present in animal corpses^22^,^28^. PVA predictions for the Pyrenean population, published in 2008, had predicted near-extinction in a time horizon of 50 years, unless non-natural adult mortality (the most sensitive vital rate in bearded vultures) would be drastically reduced^1^. In comparison, in the reintroduced Alpine population growth rate has been predicted to remain positive during the next 25 years due to a high and relatively constant adult survival probability^14^. The sole two other bearded vulture populations in Europe occur on Mediterranean islands. A small but stable population of 8–10 pairs has been inhabiting Corsica until the 1980s and 1990s^29^, but was diagnosed to face an extinction risk of 16.5% over the next 50 years, mostly due to demographic stochasticity, with pre-adult survival being the key demographic parameter obliterating future population trajectories^13^. Actually, the breeding Corsican population has dropped to 5 territorial pairs in 2014 (J.F. Seguin, pers. comm.). On Crete (Greece) 6 territories were recorded in 2014, this population also being under a high risk of extinction (S. Xirouchakis, pers. comm.).

In reintroduction and translocation programmes, it is indispensable to evaluate to which extent extractions could affect the viability of a donor population, either captive^30^ or free-ranging. Although there is an abundant literature about translocations, notably for restocking populations of exploitable game species, only few relocation studies have been motivated by pure conservation and restoration objectives^31^,^32^,^33^. The goal of this study was to assess the suitability of wild extractions for the reintroduction of a threatened, long-lived species, the bearded vulture, i.e. to assist conservation managers with evidence-based recommendations for decision making^34^,^35^.

We assessed the viability of the Pyrenean population by combining a PDP model (Population Dynamic P-system) with a Box-Benkhen design that models using the full value range (minimum and maximum) of known demographic parameters. This latter tool enables both predicting population trends and identifying the most sensitive parameters influencing population dynamics. More specifically, we 1) built a model assessing the Pyrenean population trend under different extraction scenarios while determining the relative sensitivity of key vital rates (fecundity, survival of different age classes, age at first reproduction) and accounting for density-dependent regulatory mechanisms; and 2) simulated the effects of different extraction scenarios (removal of eggs, chicks or fledglings)–foreseen for reintroducing the species in currently abandoned Spanish mountain ranges–upon the dynamics of the population serving as a donor.

## Results

**Figure 1:**
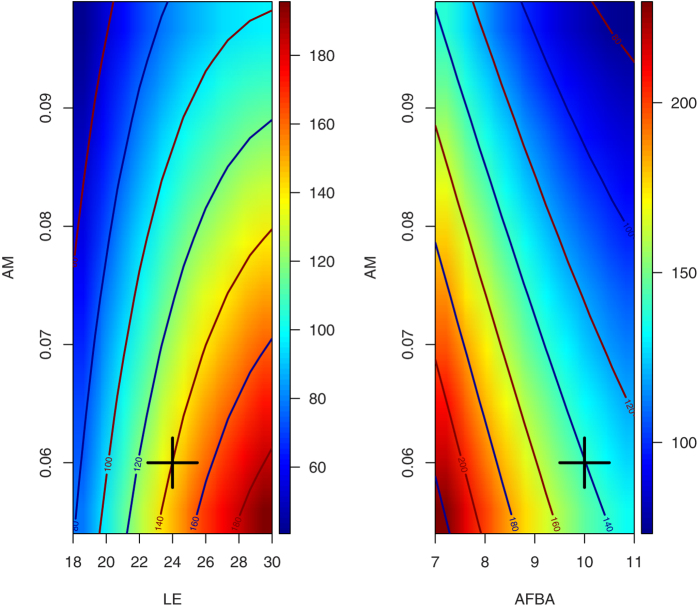
Response surface obtained with demographic parameters based on the current estimates^1^,^3^,^36^. The crosses show the current values: average life expectancy (LE): 24 years; age at first breeding attempt (AFBA): 10 years; yearly adult mortality (AM): 0.054. The colours represent the number of pairs (blue represent the lower values and red the higher).

All parameters considered (Table 1) affected the demographic trajectories in a 30 years time horizon in some way (Table 2). Moreover, there were also some significant interactions between parameters: adult mortality (AM) x average life expectancy (LE), LE x productivity (P), AM × P, AM x age of first breeding attempt (AFBA). Significant as well were the quadratic terms of LE and AFBA (Table 2; Fig. 1). Let us provide an example of how these interactions between vital rates can dramatically affect demographic trajectories. If we set the value of AM at its central point (0.054; Table 1) and productivity at its highest value (Pmax: 0.55; Table 1), the population size after a 30 years is forecasted to be 144.6 pairs (109.5 intercept + 35.10 Pmax effect). In contrast, if AM is set to its highest value (0.099) while Pmax value is kept at its central point (0.045; Table 1), the predicted population size would be only 75.94 pairs (intercept: 109–AM effect: 33.06). If both AM and Pmax are set to their maximum values (0.099 and 0.55, respectively) the projected, final population size would be 101.6 pairs (111–04–interaction effect between AM*Pmax = 9.44, Table 2).

**Figure 2:**
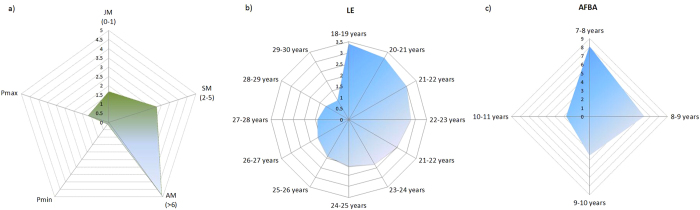
Sensitivity of the demographic parameters influencing population trends according to variation in the currently observed range values: (**a**) sensitivity of survival (expressed in percentage) with respect to age class and productivity range (JM: juvenile mortality; SM: subadult mortality; AM: adult mortaliy; P_min_ and P_max_, minimum and maximum productivity, respectively; (**b**) sensitivity variation of average life expectancy (LE, expressed in years); and (**c**) sensitivity in the age at first breeding attempt (AFBA, in years). The values presented in the vertical axes show the increase (LE, P_max_ and P_min_) or decrease (AM, SM, JM and AFBA) in the number of pairs by year.

Regarding the demographic sensitivities which were drawn from the response surface of the Box-Benhken design, in the absence of interactions we obtained a fixed value per year, whereas in case of significant interactions between parameters, the sensitivity varies over the years according to the parameter values (Fig. 2). The most sensitive parameters, i.e. those which yielded different demographic trajectories according changes in their values were (in brackets the estimated number of pair losses per year and per 1% increase in the parameter value): AM (minus 4.89 pairs/year), SM (minus 2.73 pairs/year) and JM (minus 1.69 pairs/year). Regarding AFBE and LE, projections yielded population changes ranging from an additional 2.7 (when AFBE starts at 10–11 years of age) to 8.1 pairs/year (when AFBE starts at 7–8 years). Regarding LE, the population had demographic gains ranging from 1 to 3.2 pairs/year, for scenarios in which LE increased from 29 to 30 years and from 19 to 20 years, respectively (Fig. 2).

**Figure 3:**
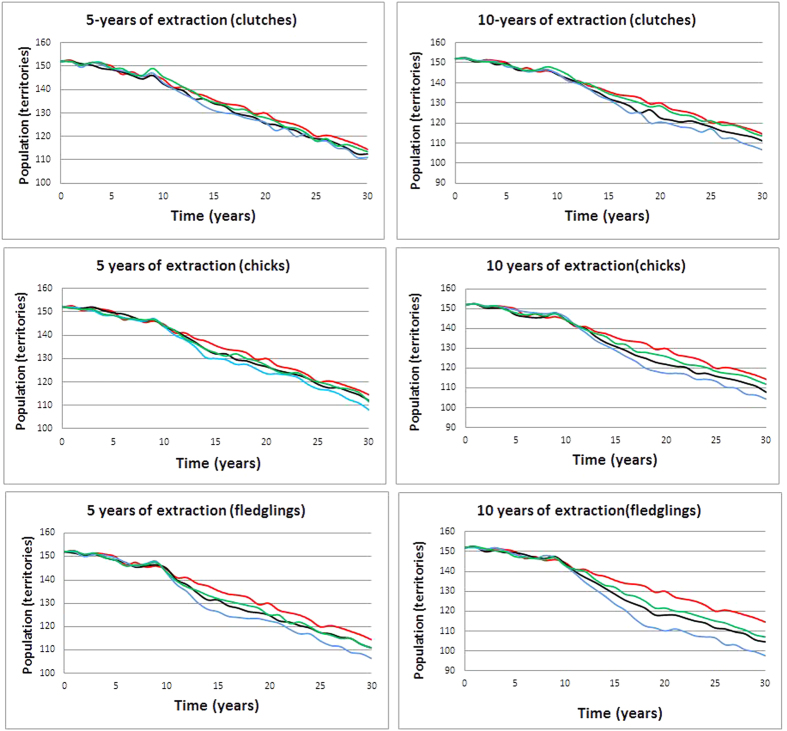
Projections of number of bearded vulture territories over time in the Pyrenees as predicted under different extraction scenarios, referring to currently estimated demographic parameters^1^,^3^. Extraction of eggs (green), nestlings (black) and fledglings (blue) vs a non-intervention scenario (red) during 5 and 10 years, respectively.

From a total of 57 projected scenarios (Table S1), 13 (22.8%) showed a positive but mostly shallow (0–2%) trend in annual population growth rate (Table 3). The projection models for population trends based on current estimates of demographic rates (Fig. 3) yielded in most cases a decline. Only combinations of P greater than 0.35, LE above 24 years, AM less than 0.06 and AFBA below or equal to 9 years would not lead to regressive scenarios (Table S1).

### Population trends under various management scenarios

We assessed population trends based on current estimates of demographic parameters^1^,^3^ after 4, 6 and 10 yearly interventions (4, 6 or 10 removals of eggs or chicks or fledglings per year) performed during 5 and 10 years (Fig. 3). Then we analysed the difference of the final population size between all scenarios and the baseline population trajectory in the absence of any intervention. The only scenario which did not differ from a non-intervention scenario was the extraction of four eggs or chicks per year in the absence of extraction of fledglings (Table 4). In the rest of the scenarios, this resulted in reductions of population size after 30 years, compared to the population size achieved by a non-intervention scenario, ranging from a loss of 2–8 pairs (egg-extraction scenario, 5–10 years), 3–10 pairs (chick extraction scenario), to a loss of 4–17 pairs (fledgling intervention scenario) (Table 4). However, in a 50 year time horizon, no extinction risk was apparent, and the final population size would range between 78–90 pairs after 5–10 years of intervention, compared to 94 pairs forecasted in a non-intervention scenario.

## Discussion

Our results suggest a reduction in the Pyrenean bearded vulture population over the next 30 years in 77% of the demographic scenarios tested. Based on this sole figure, it is thus already questionable whether extractions could at all be envisioned for future reintroduction purposes. Among the remaining 23% scenarios suggesting population increases, only one (#21) yielded an annual population growth rate greater than 2%. All other most plausible scenarios (#1, 14, 42 and 44)–based on actual empirical information about P and AFBA–would give annual growth rates from 0.04% to 1.01%. However, these scenarios would request a productivity rate higher than 0.35 (the average over the last 7 years ranged between 0.26–0.31)^3^, an age at first breeding ≤9 years (current estimated value is 10.4 years)^36^ and an adult survival of 0.946 (which is within the very top range of estimates obtained from capture-recapture models in the Pyrenees)^1^,^3^. Note that such high survival values would be similar to the estimates (0.96) obtained from the increasing, reintroduced population in the Alps^14^. Thus, these four potentially positive scenarios clearly require values that are above the current estimates obtained from the Pyrenees. In this regard, population projections based on current demographic parameters (Fig. 1) would lead to a population reduction, in agreement with previous predictions^1^. Yet, it remains open how these vital rates would develop in case of population decline, i.e. if population density would decrease, as vital rates might improve if the pressure of density-dependent regulation is relaxed.

Adult mortality commonly determines population growth in long-lived species^2^,^12^,^37^,^38^. For both the Alpine^14^ and Pyrenean^1^ bearded vulture populations it has been established that adult mortality is the demographic rate with the highest sensitivity. Adult mortality has been steadily increasing over the recent years in the Pyrenean population, and demographic forecasts, including the present study, predict a negative trend and its near-extinction over the next 50 years if non-natural mortality (i.e. illegal poisoning which greatly affects the adult segment of the population) continues unabated^39^.

The age at first breeding attempt has been steadily increasing in the Pyrenean population, passing from 8 years during the period 1987–2006^36^ to 10 years presently^40^. Given the progressive increase in breeding density over recent decades, this fact is most likely the outcome of density-dependent regulation. In parallel, there was an increase in the fraction of non-territorial but potential breeders (i.e. mature floaters) in the population, from 39% in 1987–2006^40^ to 68% today (personal unpublished information). All in all, this reinforces the hypothesis that the population has reached saturation as already inferred from productivity trend and changes in mating system (recent increase in the proportion of polyandrous territories)^41^, as well as in the distance between neighbouring nests that declined by >20% between 1992 and 2002^42^.

There is an apparent contradiction between the regressive predictions^1^ and the population growth which has been observed in the Pyrenees during the last decade^42^,^43^. No changes have been operated in feeding schemes, hence population growth cannot be attributed to enhanced preadult survival due to increased artificial food supply^16^,^17^,^44^,^45^. Regarding wild ungulate populations they have increased in size^44^,^46^. We thus attribute the increase in population size to a higher adult survival in response to a reduction of poisoning (baits to fight carnivores) and lead intoxication (hunting ammunition)^22^,^28^. From 1988–2006 to 2006–2011 adult survival seems to have increased from 87.8%^1^ to 94.6%^3^. However, according to our simulations which predict negative trends in 77% of the scenarios tested, there is still a risk of facing demographic degradation into the future if efforts to combat these negative drivers are released and if new anthropogenic sources of mortality emerge. In this respect, the recent approval of diclofenac in Spain represents a major novel source of concern^47^ given that this veterinary drug has almost exterminated the populations of several Asian vulture species over wide areas^48^,^49^. The future survival of the Pyrenean bearded vulture population will thus depend on our ability to mitigate any factors negatively affecting the most sensitive demographic parameters such as adult survival.

Our simulations show that most egg, chick and fledgling extraction scenarios to reinforce or reintroduce this long-lived species in particular, and by extension all long-lived species in general, may have detrimental effects on the population dynamics of the donor population. This finding corroborates other studies that have demonstrated that selective harvesting can indirectly reduce recruitment, thereby potentially impacting population growth rate^50^. Our simulations furthermore show that the effects of extractions will not be detectable until more than nine years after the initiation of such interventions. Under most circumstances, these interventions would imply significantly smaller final population sizes, compared to non-intervention scenarios, this even under shallow extraction schemes (typically egg removal from more than 4 nests during 5 years). Given the residual risks due to model uncertainty, however, we advise to implement only sensible extraction schemes if at all, applying here the principle of precaution. In effect, egg extraction bears a great risk of failure due to the low hatching success observed today in the wild (25–45%; A. Margalida, unpubl. data), meaning that only a tiny fraction of extracted eggs are likely to be viable. Indeed, alternative, less risky management options already exist for the bearded vulture such as the collection of only the second, freshly-born chick from double clutches (given that in this species siblicide is the rule)^51^,^52^,^53^ and the release from captive stock^14^. Finally, among non-invasive approaches, targeted supplementary feeding at the limits of the current Pyrenean distribution range, while stopping artificial feeding in the core population area, would contribute to boost dispersal and future settlement into neighbouring mountain ranges^42^. The simulations presented here offer enough evidence to assist managers and policy-makers for adopting the most rigorous and effective restoration operations for the conservation of this rare emblematic raptor^35^.

## Methods

### Data collection and parameter estimates

The Pyrenean (Spain and France) bearded vulture population has been intensively monitored within the framework of the Species’ Recovery Plan of the Spanish Regional Governments of Basque Country, Navarra, Aragón and Catalonia, as well of the French Pyrenean Departments. Field surveys to monitor annual population size and breeding performance have been carried out since the 1980´s, while survival probabilities have been estimated from capture-mark-resighting^1^,^3^,^27^,^54^.

Between 1994 and 2011, all known Pyrenean territories were visited several times monthly to search for signs of occupancy (territorial and/or courtship activity, nest arrangement/building), and to record reproductive parameters. Productivity (number of fledglings per pair–or breeding unit in the case of polyandrous trios‐and year) was also estimated^3^. The ranges of the observed parameters were used to feed our models, assuming an even sex ratio at birth^12^,^13^. From 1987 to 2011, a total of 106 individuals were radio- or satellite-tracked (R. Heredia, FCQ and A.M. argalida unpubl. data), which allowed estimating survival^1^,^3^. We distinguished three age classes according to the most parsimonious age-model obtained in previous analyses^1^,^3^: juveniles (0–1 year old), subadults (2–5 years old) and adults (>6 years old). Given that demographic rates have decreased over the years^3^, we considered the range of values obtained during the last 5 years (2007–2011), referring to various demographic studies (Table 1 and Table S1).

All bearded vultures found dead (radio-tagged or not) were investigated to determine the cause of death^22^. In modelling, maximum population size (i.e. carrying capacity *K*), has been set to 300 breeding individuals in the Pyrenees, which is twice the current breeding population size, based on estimates of natural (i.e. without artificial feeding) food biomass and suitable habitat availability^17^,^18^,^44^.

That density-dependence negatively impacts productivity has already been demonstrated for the study population^27^. In the simulations we considered negative density-dependence effects on productivity, applying the equation used in *Vortex*: 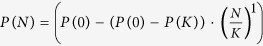 where *P*(*N*) is the percentage of females that breed at population size *N*, *P*(*N*) the percentage breeding when the population is at carrying capacity (*K*), and *P*(0) the percentage breeding when the population is close to zero. Although density-dependent effects on productivity are thought to be the principal factor of population regulation, recent surveys suggest that other vital rates may also be affected, such as the fraction of the population that breeds in a given year, which has also declined over time. Note that the reliance on the whole range of parameter values observed somehow already encapsulates within demographic projections any variations linked to density-dependence effects.

### Population modelling

For studying the effects of varying vital rates on population trends we used a surface response model (Box-Behnken) with seven factors corresponding to the demographic parameters (Table 1) and four repetitions of the central point used to estimate the variability (these four repetitions refer to one single given scenario), totalling 60 “experiments” that corresponds to 57 different scenarios (Table S1). These “experiments” were performed using PDP models^55^.

### PDP models

PDP models are biocell-inspired models that attempt to imitate cell behaviour^55^,^56^. PDP models work in parallel and are able to model complex processes^19^,^56^,^57^,^58^. They are formed by four components: number of environments, membrane structure, initial alphabet and evolution rules^55^.

Here we used two PDP models (density-dependent model; hereafter DDM; and extraction model, hereafter EM). Both allow simulating the bearded vulture population under different scenarios. In the DDM we took into account variations in productivity according to population size. In both models the environmental stochasticity has not been considered. In the EM we subdivided productivity variables into subcategories (percentages of pairs with egg laying, hatching success and fledging success) in order to account for the effects of different intervention scenarios (eggs, chicks or fledgling removal) on productivity and population size. The structure of both models is similar and there are differences mainly in the rules of the reproduction process (with or without intervention scenarios, see Supplementary Information).

As our study area comprises the entire Pyrenees, we considered only one environment in both models. We kept the membrane structure as simple as possible^54^, consisting of two membranes: *μ* = [[ ]_1_]_0_. The initial alphabet in the case of DDM is 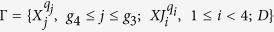. For the EM model it is necessary to add the objects 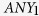, 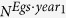, 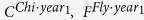, where the object 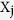 is associated with the individuals achieving reproductive age, while 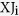 is associated with non-breeding individuals. The object *D* allows the generation of objects that are used for the control of maximum carrying capacity. 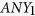 allows for the control of annual interventions (see below) and the objects *N*, *C* and F allow for the performance of the interventions. The evolution rules of the model are detailed in Supplementary Information.

### Box-Behnken design

The response surface methodology has been widely used in the domain of food research to establish a functional relationship between the response variable and the explanatory variables involved^59^. We used a response surface approach to estimate population size of bearded vultures in the Pyrenees (DDM) in a time horizon of 30 years, depending on the values of the parameters considered (Table 1). The response surface designs are a subset of experimental designs used to model the relationship between the independent variables or factors 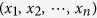 and the response variable^59^, using linear models, quadratic or even higher order models. The Box-Behnken design^59^ is usually more efficient in terms of number of “experiments” and is rotatable (or near rotatable) such that it estimates the coefficients of the first and second most efficient order. The results were analyzed using DOE.base, a package of the R program (16 R 2.10.1).

### Modelling management intervention scenarios

Using the EM, we estimated the effects that sustained extraction of eggs, chicks or fledglings can have on the population trend of the Pyrenean bearded vulture in a 30 years time horizon. Model outcome was compared to a base scenario of non-intervention, accounting for the demographic rates used in the model (Table 1). Three types of interventions were simulated: two related to nest management (removing the eggs of a clutch or the chick) during the incubation and rearing phase, respectively; one during the post-fledging phase (trapping of a young after fledging). Based on our own recent field observations, the annual removal of 4–10 clutches, 4–10 chicks or 4–10 fledglings, on average, would affect as much as 4–11% of egg layings, 6–15% of hatched chicks and 10–25% of fledglings, respectively, of the Pyrenean bearded vulture population. We considered two hypothetical, but realistic scenarios in which extractions would be performed during 5 and 10 years, respectively. Extraction scenarios were modelled with respect to the three following breeding phases: First phase: incubation; extraction of four, six and 10 clutches, respectively; although clutches consist of two eggs in the Bearded vulture, only one chick survives^49^, meaning that the breeding outcome will always be one chick or failure. Moreover, not all eggs hatch (only 75% hatched during the five last years of the study), which should be accounted for while estimating the probability that extracted eggs are fertile. Second phase: chick-rearing; removal of 4, 6 and 10 chicks, respectively, directly from the nest. Third phase: post-fledging; capture and extraction of 4, 6 and 10 fledglings, respectively, once they have left the nest.

## Additional Information

**How to cite this article**: Margalida, A. *et al.* Assessing the impact of removal scenarios on population viability of a threatened, long-lived avian scavenger. *Sci. Rep.* **5**, 16962; doi: 10.1038/srep16962 (2015).

## Supplementary Material

Supplementary Information

## Figures and Tables

**Table 1:** Range (minimum-maximum) values of demographic parameters obtained from own empirical data and peer-reviewed published information (for details, see Methods) used to calculate the viability of the Pyrenean bearded vulture population.

Parameters*	Minimum	Maximum
LE	18	30
AFBA	7	11
JM (0–1 year)	0.017	0.047
SM (2–5 years)	0.091	0.11
AM (>6 years)	0.054	0.099
Pmin	0.08	0.12
Pmax	0.35	0.55

LE: average life expectancy; AFBA: age at first breeding attempt; JM: juvenile mortality; SM: subadult mortality; AM: adult mortality; Pmin: minimum productivity; Pmax: maximum productivity.

^*^All parameters are the average estimates obtained from 2006–2011 (Margalida *et al.*, 2014) except for life expectancy obtained from Oro *et al.*, (2008) and own data.

**Table 2:** Coefficient values of the response surface and significance level of the variables and interactions that were tested in the analysis of variance.

Parameter	Value	p-value
Intercept	109.0	<0.0001
^(1)^LE	39.50	<0.0001
^(1)^AFBA	−32.42	<0.0001
^(1)^JM	−7.65	<0.0001
^(1)^SM	−7.79	<0.0001
^(1)^AM	−33.06	<0.0001
^(1)^PMax	35.10	<0.0001
^(1)^LE*AM	−14.69	<0.0001
^(1)^LE*Pmax	8.19	0.0089
^(1)^AFBA*Pmin	−8.81	0.0053
^(1)^AFBA*Pmax	−16.19	<0.0001
^(1)^AFBA*AM	7.67	0.01337
^(1)^AM*Pmax	−9.44	0.0032
^(1)^(LE)^2^	−12.06	<0.0001
^(1)^(AFBA)^2^	10.69	0.00013

^(1)^
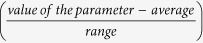
.

**Table 3:** Scenarios with a combination of demographic parameters in which the population trend after 30 years yield more than the current number of territories (n = 152).

Scenario	LE	AFBA	JM	SM	AM	P	PS	PI
**1**	**24**	**9**	**0.032**	**0.091**	**0.054**	**0.358**	**154**	**0.04%**
5	24	9	0.032	0.091	0.054	0.385	157	0.10%
49	24	7	0.017	0.1005	0.0765	0.351	158	0.13%
23	24	7	0.032	0.1005	0.099	0.351	162	0.22%
53	24	7	0.017	0.1005	0.0765	0.356	163	0.23%
37	24	9	0.017	0.091	0.0765	0.389	163	0.24%
30	30	7	0.032	0.11	0.0765	0.374	168	0.34%
**14**	**30**	**9**	**0.032**	**0.1005**	**0.0765**	**0.361**	**176**	**0.53%**
16	30	9	0.032	0.1005	0.0765	0.377	178	0.57%
**44**	**30**	**9**	**0.047**	**0.1005**	**0.054**	**0.359**	**185**	**0.72%**
26	30	7	0.032	0.091	0.0765	0.377	194	0.92%
**42**	**30**	**9**	**0.017**	**0.1005**	**0.054**	**0.354**	**198**	**1.01%**
21	24	7	0.032	0.1005	0.054	0.352	271	2.60%

The scenarios are numbered with respect to final population size (PS and PI) in increasing order from minimum to maximum population increases. The most plausible scenarios, i.e. scenarios in accordance with currently observed AFBA and P are shown in bold type. PS: final population size; PI: percentage of population increase. For other abbreviations, see Table 1.

**Table 4:** Population size (number of territories) of the bearded vulture after 30 years of different extraction scenarios for either 5 or 10 years, in comparison to a non-intervention scenario.

Time-period (years)	Remove	5-years difference with respect to no intervention	Population size after 5 years of interventions	10-years difference with respect to no intervention	Population size after 10 years of interventions
Clutches	4	−1	114	−1	114
	6	−2	113	−4	111
	10	−4	111	−8	107
Chicks	4	−3	112	−3	112
	6	−3	112	−7	108
	10	−7	108	−10	105
Fledglings	4	−4	111	-8	107
	6	−4	111	−10	105
	10	−8	107	−17	98
