# Supplementary material for: Assessing the impact of removal scenarios on population viability of a threatened, long-lived avian scavenger
Source: Sci Rep. 2015 Nov 23;5:16962. doi: 10.1038/srep16962 (PMC4655339; doi:10.1038/srep16962)
Supplement: Supplementary Information [file srep16962-s1.pdf]

SUPPLEMENTARY INFORMATION FOR:

Assessing the impact of removal scenarios on population viability of a threatened, long-lived avian scavenger

Antoni Margalida, MªÀngels Colomer, Daniel Oro, Raphaël Arlettaz &
José A. Donázar

Evolution rules

Extraction model (EM)

The proposed PDP model is structured in eight sequenced modules, corresponding to basic biological processes, as are mortality and reproduction. The latter process was broken down into laying, hatching and chick-rearing in order to estimate the effect of the intervention at various stages. The feeding process was not modeled, because it is assumed that food resources are not a limiting factor (see Margalida et al., 2011, Margalida & Colomer 2012). Each module is a step in the model, the complete execution of the loop is eight steps that correspond to a period of one year. Below we describe the rules that apply to each of the eight steps. The parameters used in the model rules appear in Table S1.

Step 1. The central process is natural mortality, although the aim of the first rule is to generate objects that subsequently allow monitoring of the maximum density of animals in the area.

$$r_1 \equiv D[\quad]_1^0 \longrightarrow R_1, [a^{0.9d}, e^{0.2d}]_0^0.$$

The objects a will allow for the control of the maximum load, while objects e are used to generate randomness in population size. R_1 is a counter that evolves at

each step and is used to synchronize the model. According to the probability of death that depends on the age of the individuals, the same objects evolve while others disappear, if the animal dies.

$$r_2 \equiv \left[XJ_j \xrightarrow{m_1} \#\right]_0^0, 1 \leq j \leq g_1.$$

$$r_3 \equiv \left[XJ_j \xrightarrow{1-m_1} Y_j\right]_0^0, 1 \leq j \leq g_1.$$

$$r_4 \equiv \left[XJ_j \xrightarrow{m_2} \#\right]_0^0, g_1 < j \leq g_2.$$

$$r_5 \equiv \left[XJ_j \xrightarrow{1-m_2} Y_j\right]_0^0, g_1 < j \leq g_2.$$

$$r_6 \equiv \left[XJ_j \xrightarrow{m_3} \#\right]_0^0, g_2 < j < g_4.$$

$$r_7 \equiv \left[XJ_j \xrightarrow{1-m_3} Y_j\right]_0^0, g_2 < j < g_4.$$

$$r_8 \equiv \left[X_j \xrightarrow{m_3} \#\right]_0^0, g_4 \leq j < g_3.$$

$$r_9 \equiv \left[X_j \xrightarrow{1-m_3} Y_j\right]_0^0, g_4 \leq j < g_3.$$

$$r_{10} \equiv \left[X_{g_3} \longrightarrow \#\right]_0^0.$$

Step 2 The central objective of this step is to start the process of reproduction with egg laying; as occurred in the first step here are applied in parallel rules unrelated to the process of reproduction, such as rules 11 and 12.These rules generate the model randomness in the final population size after reaching maximum density, the 50% objects type e are dissolved while the remaining objects type a evolves.

$$r_{11} \equiv \left[e \xrightarrow{0.5} a\right]_1^0.$$

$$r_{12} \equiv \left[e \xrightarrow{0.5} \#\right]_1^0.$$

The objects associated with individuals of reproductive age that breed successfully generate news objects, EG representing laying eggs. The objects Y_j associated with animals evolve to objects Z_j.

$$r_{13} \equiv \left[Y_j \xrightarrow{0.5\cdot SB} Z_j, EG\right]_0^0, g_4 \leq j \leq g_5.$$

$$r_{14} \equiv \left[Y_j \xrightarrow{1-0.5\cdot SB} {}_j\right]_0^0, g_4 \leq j \leq g_5.$$

$$r_{15} \equiv \left[Y_j \longrightarrow Z_j\right]_0^0, 1 \leq j < g_4.$$

$$r_{16} \equiv \left[Y_j \longrightarrow Z_j\right]_0^0, g_5 < j \leq g_3.$$

$$r_{17} \equiv [R_1 \longrightarrow R_2]_0^0.$$

Step 3 Nest interventions: Clutches

There are many objects N as nests intervened.

$$r_{18} \equiv [EG, N \longrightarrow \#]_0^0.$$

$$r_{19} \equiv R_2[\quad]_1^0 \longrightarrow R_3[\quad]_1^+.$$

Step 4 Hatching success

Clutches can be double but only a chick can complete the process successfully; we can thus establish the relationship of one egg for each laying. Some of the eggs will hatch (pb), which generated hatched objects Z_0 associated with new individuals entering the inner membrane labeled with the value 1.

$$r_{20} \equiv EG[\quad]_1^+ \xrightarrow{pb} [Z_0]_1^+.$$

$$r_{21} \equiv EG[\quad]_1^+ \xrightarrow{1-pb} [\#]_1^+.$$

All other objects associated with bearded vulture also come into the inner membrane, as well as the C and F objects. These objects store information of the interventions. If N objects left over are dissolved because its function has ended.

$$r_{22} \equiv Z_j[\quad]_1^+ \longrightarrow \left[Z_j\right]_1^+, 1 \leq j < g_3.$$

$$r_{23} \equiv C[\quad]_1^+ \longrightarrow [C]_1^+.$$

$$r_{24} \equiv F[\quad]_1^+ \longrightarrow [F]_1^+.$$

$$r_{25} \equiv N[\quad]_1^+ \longrightarrow [\#]_1^+.$$

$$r_{26} \equiv [R_3 \longrightarrow R_4]_0^0.$$

Step 5 Nests intervention: chicks in the nest

The number of intervened nests is C, the removed chicks at nest will disappear.

$$r_{27} \equiv [Z_0, C]_1^+ \longrightarrow [\ \ ]_1^0.$$

The object Fthat will allow modeling fledgling interventions evolves as not consumed, the purpose of the evolution of this object is to prevent the rules of intervention for the fledglings are applied in the wrong time.

$$r_{28} \equiv [F]_1^+ \longrightarrow F'[\ \ ]_1^0.$$

$$r_{29} \equiv R_4[\ \ ]_1^+ \longrightarrow R_5[\ \ ]_1^0.$$

Step 6. Several of the hatched chicks abandon the nest successfully. The central objective of this step is to model the fledglings.

$$r_{30} \equiv [Z_0]_1^0 \xrightarrow{pf} [Y_0]_1^-.$$

$$r_{31} \equiv [Z_0]_1^0 \xrightarrow{1-pf} [\#]_1^-.$$

In parallel to the process of fledglings are applied rules that increment by one the age of the animals while controlling maximum carrying capacity.

$$r_{32} \equiv \left[Z_j, a\right]_1^0 \longrightarrow X'_{j+1}[\ \ ]_1^-,\ g_4 - 1 \leq j < g_3.$$

$$r_{33} \equiv \left[Z_j\right]_1^0 \longrightarrow XJ'_{j+1}[\ \ ]_1^-, 1 \leq j < g_4 - 1.$$

$$r_{34} \equiv F'[\ \ ]_1^0 \longrightarrow [F']_1^-.$$

$$r_{35} \equiv R_5[\ \ ]_1^0 \longrightarrow R_6[\ \ ]_1^-.$$

Step 7 Fledglings

Fledglings as there are objects F' of the type are extracted, these fledglings disappear ecosystem.

$$r_{36} \equiv [Y_0, F']_1^- \longrightarrow [\#]_1^0.$$

Unconsumed objects will be dissolved

$$r_{37} \equiv [a]_1^- \longrightarrow [\#]_1^0.$$

$$r_{38} \equiv \left[Z_j\right]_1^- \longrightarrow [\#]_1^0, 1 \leq j < g_3.$$

$$r_{39} \equiv [C]_1^- \longrightarrow [\#]_1^0.$$

Evolution objects that are associated with the vulture, this evolution allows objects not start the cycle prematurely

$$r_{40} \equiv \left[X'_j\right]_1^- \longrightarrow \left[X''_j\right]_0^0, g_4 \le j \le g_3.$$

$$r_{41} \equiv \left[XJ'_j\right]_1^- \longrightarrow \left[XJ''_j\right]_0^0, 1 \le j < g_4.$$

$$r_{42} \equiv R_6[\quad]_1^- \longrightarrow R_7[\quad]_1^0.$$

Step 8 Update

Restoring the original configuration, the system is prepared to start the simulation of the following year, i.e., restart the loop.

$$r_{43} \equiv [F']_1^0 \longrightarrow [\#]_1^0.$$

$$r_{44} \equiv [Y_0]_1^0 \longrightarrow XJ_1[\quad]_1^0.$$

$$r_{45} \equiv \left[X''_j \longrightarrow X_j\right]_0^0, g_4 \le j \le g_3.$$

$$r_{46} \equiv \left[XJ''_j \longrightarrow XJ_j\right]_0^0, 1 \le j < g_4.$$

$$r_{47} \equiv \left[R_7, Y_j \longrightarrow Y_{j+1}, D, N^{Egs \cdot year_{j+1}}, C^{Chi \cdot year_{j+1}}, F^{Fly \cdot year_{j+1}}\right]_0^0, 1 \le j < yearsim.$$

Density-dependent model (DDM)

The proposed PDP model is structured in four sequenced modules: mortality, count number of adult animals, reproduction and restore initial configuration.

Step 1 Generation of objects for controlling the maximum load and mortality rules

$$r_1 \equiv D[\quad]_1^0 \longrightarrow R_1[a^{d \cdot 0.9}\, e^{d \cdot 0.2}]_1^0 .$$

$$r_2 \equiv \left[XJ_j \xrightarrow{m_1} \#\right]_0^0, 1 \le j \le g_1.$$

$$r_3 \equiv \left[XJ_j \xrightarrow{1-m_1} Y_j\right]_0^0, 1 \le j \le g_1.$$

$$r_4 \equiv \left[XJ_j \xrightarrow{m_2} \#\right]_0^0, g_1 < j \le g_2.$$

$$r_5 \equiv \left[XJ_j \xrightarrow{1-\ m_2} Y_j\right]_0^0, g_1 < j \leq g_2.$$

$$r_6 \equiv \left[XJ_j \xrightarrow{m_3} \#\right]_0^0, g_2 < j < g_4.$$

$$r_7 \equiv \left[XJ_j \xrightarrow{1-m_3} Y_j\right]_0^0, g_2 < j < g_4.$$

$$r_8 \equiv \left[X_j \xrightarrow{m_3} \#\right]_0^0, g_4 \leq j < g_3.$$

For each adult that survive it's generate one object type *count*

$$r_9 \equiv \left[X_j \xrightarrow{1-m_3} Y_j, count_1\right]_0^0, g_4 \leq j < g_3.$$

$$r_{10} \equiv \left[X_{g_3} \longrightarrow \#\right]_0^0.$$

Step 2 to 14 Count the adult animals

Evolution of objects that allow control of maximum load

$$r_{11} \equiv \left[e \xrightarrow{0.5} a\right]_1^0.$$

$$r_{12} \equiv \left[e \xrightarrow{0.5} \#\right]_1^0.$$

Count number of adult animals

$$r_{13} \equiv \left[count_j\ count_i \longrightarrow count_{j+i}\right]_1^0, 1 \leq j \leq \frac{d}{2}, 1 \leq i \leq d.$$

$$r_{14} \equiv [\ R_i \longrightarrow R_{i+1}]_0^0, 1 \leq i \leq 13.$$

$$r_{15} \equiv R_{14}[\quad]_1^0 \longrightarrow R_{15}[\quad]_1^-.$$

Step 15 maximum load control and preparing to start playback settings

$$r_{16} \equiv \left[count_j\right]_1^- \longrightarrow count_j^j[\quad]_1^+, 1 \leq j \leq d.$$

$$r_{17} \equiv [YY_i]_1^- \longrightarrow Y_i[\quad]_1^+, 1 \leq i < g_4.$$

$$r_{18} \equiv [YY_i\ a]_1^- \longrightarrow Y_i[\quad]_1^+, g_4 \leq i < g_3.$$

$$r_{19} \equiv R_{15}[\quad]_1^- \longrightarrow R_{16}[\quad]_1^+.$$

Step 16 Reproduction rules

$$r_{20} \equiv \left[YY_j\ \right]_1^+ \longrightarrow [\quad]_1^0, g_4 \leq j < g_3.$$

$$r_{21} \equiv \left[Y_j \; count_i \xrightarrow{0.5\cdot\left(Maxf-(Maxf-Minf)\cdot\frac{i}{d}\right)} Z_j \;\; Z_0^k \right]_0^0, g_4 \le j \le g_5, 1 \le i \le d.$$

$$r_{22} \equiv \left[Y_j \; count_i \xrightarrow{1-\,0.5\cdot\left(Maxf-(Maxf-Minf)\cdot\frac{i}{d}\right)} Z_j \right]_0^0, g_4 \le j \le g_5, 1 \le i \le d.$$

$$r_{23} \equiv \left[Y_j \longrightarrow Z_j \right]_0^0, 1 \le j < g_4.$$

$$r_{24} \equiv \left[Y_j \longrightarrow Z_j \right]_0^0, g_5 < j \le g_3.$$

$$r_{25} \equiv [a]_1^+ \longrightarrow [\;\;]_1^0.$$

$$r_{26} \equiv [R_{16} \longrightarrow R_{17}]_0^0.$$

Step 17 Restore initial configuration

$$r_{27} \equiv [Z_0 \longrightarrow XJ_1]_0^0.$$

$$r_{28} \equiv \left[Z_j \longrightarrow X_{j+1} \right]_0^0, g_4 - 1 \le j < g_3.$$

$$r_{29} \equiv \left[Z_j \longrightarrow XJ_{j+1} \right]_0^0, 1 \le j < g_4 - 1.$$

$$r_{30} \equiv [R_{17} \longrightarrow D]_0^0.$$

References

Margalida, A. & Colomer, M.A. Modelling the effects of sanitary policies on European vulture conservation. *Sci. Rep.* **2**, 753 (2012)

Margalida, A., Colomer, M.A. & Sanuy, D. Can wild ungulate carcasses provide enough biomass to maintain avian scavenger populations? An empirical assessment using a bio-inspired computational model. *PLoS ONE* **6**, e20248 (2011).

Table S1. Scenarios with combinations of demographic parameters in which the population size after 30 years would be below 152 territories. The scenarios are presented from minimum to maximum population decreases. LE: average life expectancy; AFBA: age at first breeding attempt; JM: juvenile mortality; SM: subadult mortality; AM: adult mortality; Fmin: minimum fecundity; Fmax: maximum fecundity; PI: relative population decrease.

Scenario	LE	AFBA	JM	SM	AM	Fmin	Fmax	PI
55	24	7	0.047	0.1005	0.0765	0.12	0.361	-0.02%
38	24	9	0.047	0.091	0.0765	0.1	0.368	-0.09%
22	24	11	0.032	0.1005	0.054	0.1	0.375	-0.14%
39	24	9	0.017	0.11	0.0765	0.1	0.373	-0.16%
50	24	11	0.017	0.1005	0.0765	0.08	0.369	-0.22%
51	24	7	0.047	0.1005	0.0765	0.08	0.387	-0.25%
6	24	9	0.032	0.11	0.054	0.12	0.375	-0.29%
2	24	9	0.032	0.11	0.054	0.08	0.370	-0.36%
17	24	7	0.032	0.1005	0.054	0.1	0.389	-0.42%
40	24	9	0.047	0.11	0.0765	0.1	0.378	-0.54%
28	30	11	0.032	0.091	0.0765	0.1	0.331	-0.81%
57	24	9	0.032	0.1005	0.0765	0.1	0.354	-0.88%
25	18	7	0.032	0.091	0.0765	0.1	0.375	-0.93%
32	30	11	0.032	0.11	0.0765	0.1	0.346	-1.06%
12	30	9	0.032	0.1005	0.0765	0.12	0.379	-1.09%
46	30	9	0.017	0.1005	0.099	0.1	0.376	-1.10%
10	30	9	0.032	0.1005	0.0765	0.08	0.362	-1.23%
48	30	9	0.047	0.1005	0.099	0.1	0.379	-1.26%
7	24	9	0.032	0.091	0.099	0.12	0.368	-1.40%

33	24	9	0.017	0.091	0.0765	0.1	0.386	-1.41%
29	18	7	0.032	0.11	0.0765	0.1	0.359	-1.43%
41	18	9	0.017	0.1005	0.054	0.1	0.380	-1.46%
54	24	11	0.017	0.1005	0.0765	0.12	0.360	-1.47%
15	18	9	0.032	0.1005	0.0765	0.12	0.374	-1.49%
18	24	11	0.032	0.1005	0.054	0.1	0.371	-1.49%
3	24	9	0.032	0.091	0.099	0.08	0.354	-1.49%
13	18	9	0.032	0.1005	0.0765	0.08	0.357	-1.50%
56	24	11	0.047	0.1005	0.0765	0.12	0.375	-1.58%
34	24	9	0.047	0.091	0.0765	0.1	0.366	-1.59%
24	24	11	0.032	0.1005	0.099	0.1	0.363	-1.60%
52	24	11	0.047	0.1005	0.0765	0.08	0.371	-1.61%
8	24	9	0.032	0.11	0.099	0.12	0.368	-1.66%
35	24	9	0.017	0.11	0.0765	0.1	0.375	-1.70%
19	24	7	0.032	0.1005	0.099	0.1	0.389	-1.72%
4	24	9	0.032	0.11	0.099	0.08	0.376	-1.73%
43	18	9	0.047	0.1005	0.054	0.1	0.358	-1.73%
36	24	9	0.047	0.11	0.0765	0.1	0.353	-1.86%
45	18	9	0.017	0.1005	0.099	0.1	0.364	-2.29%
20	24	11	0.032	0.1005	0.099	0.1	0.375	-2.37%
47	18	9	0.047	0.1005	0.099	0.1	0.349	-2.41%
11	18	9	0.032	0.1005	0.0765	0.12	0.376	-2.47%
27	18	11	0.032	0.091	0.0765	0.1	0.384	-2.48%
9	18	9	0.032	0.1005	0.0765	0.08	0.369	-2.50%
31	18	11	0.032	0.11	0.0765	0.1	0.369	-2.55%

Table S2. Description of the variables used in the model.

d maximum density (carrying capacity)
m_1 juvenile mortality
m_2 subadult mortality
m_3 adult mortality
g_1 juvenile life expetancy
g_2 subadult life expentacy
g_3 adult life expentancy
g_4 age first breeding attempt
g_5 age of last breeding attempt (senescence)
SB probability that a pair in reproductive age start laying
pb probability that a egg hatch with success
pf probability that a hatched chick abandon the nests successfully
Egs removal of eggs
Chi removal of chicks
Fly removal of fledglings
year$_1$ when exist intervention and 0 when not intervention take place
$Maxf$ maximum productivity
$Minf$ minimum productivity
